# Prognostic value of angiopoietin-2 in non-small cell lung cancer patients: a meta-analysis

**DOI:** 10.1186/s12957-016-0992-4

**Published:** 2016-09-02

**Authors:** Zi-Xue Xuan, Su Zhang, Shou-Jun Yuan, Wei Wang, Jia Yu

**Affiliations:** 1 Department of Pharmacy, Zhejiang Provincial People’s Hospital, Hangzhou, 310014 China; 2 Department of Pharmacology and Toxicology, Beijing Institute of Radiation Medicine, Beijing, 100850 China; 3 Department of Pharmacy, Zhejiang Medical College, Hangzhou, 310053 China

**Keywords:** Non-small cell lung cancer, NSCLC, Angiopoietin-2, Ang-2, Prognosis

## Abstract

**Background:**

Non-small cell lung cancer (NSCLC) is the most frequent cause of cancer deaths worldwide. The targeted therapy had made important progress in recent years, but few potential predictive biomarkers for prognosis of NSCLC patients were identified. Angiopoietin-2 (Ang-2), a cytokine upregulated in tumor endothelial cells and some tumor cells including NSCLC, is a partial agonist and antagonist of angiopoietin-1 (Ang-1). Ang-1 is another ligand for the tyrosine kinase receptor Tie2; it promotes recruitment of pericytes and smooth muscle cells, stabilizing vascular networks by binding to Tie2. Although many studies mainly considered that Ang-2 correlated with progression and prognosis of NSCLC significantly, there are much conflicting and controversial data. Therefore, we conducted a meta-analysis to assess the relationship between Ang-2 and prognosis, a clinical outcome of NSCLC.

**Methods:**

The search was based on major databases from PubMed, Cochrane Library, EMBASE, and CNKI, and 20 eligible publications (range from 2002 to 2015) are included in our meta-analysis with 2011 NSCLC patients in total. These studies illuminated the correlation between the expression of Ang-2 and NSCLC, based on either prognostic factors or clinicopathological features. Pooled calculations were carried out on the odds ratio (OR) and the corresponding 95 % confidence interval (CI) to perform this meta-analysis, and all statistical analyses were carried out by STATA 12.0 and Review Manager 5.3.

**Results:**

According to our results, the expression of Ang-2 in NSCLC tissues was significantly higher than that in normal lung tissues, indicating that Ang-2 over-expression may be a predictive marker (pooled OR = 5.09, corresponding 95 % confidence interval (95 % CI) 3.10–8.36, *p* = 0.000). In addition, our pooled data showed that Ang-2 expression was positively correlated with tumor stages (pooled OR = 3.58, 95 % CI 2.40–5.35, *p* = 0.000), differentiation (pooled OR = 0.65, 95 % CI 0.45–0.94, *p* = 0.02), lymphatic invasion (pooled OR = 3.15, 95 % CI 1.97–5.03, *p* = 0.000), and poor survival (pooled OR = 1.93, 95 % CI 1.47–2.52, *p* = 0.000) of NSCLC, but seems to have no significant impact on tumor size (pooled OR = 1.09, 95 % CI 0.59–2.00, *p* = 0.78).

**Conclusions:**

These results demonstrate that Ang-2 expression significantly correlated with poor prognosis for patients with NSCLC.

## Background

Lung cancer is the leading cause of cancer deaths worldwide, whose 5-year survival rate is only 16 % [1]. In China, nearly 560,000 people die because of lung cancer; unfortunately, the incidence and mortality of lung cancer is still on the rise, especially non-small cell lung cancer (NSCLC), which makes up 80 % of lung cancer [2]. Due to the emergence of inhibitors of epidermal growth factor receptors (EGFRs), anaplastic lymphoma kinase (ALK) and other targeted drugs, the survival of patients improved greatly [3–5]. Although the targeted therapy of NSCLC makes important progress, efforts to identify new additional prognostic and predictive biomarkers for NSCLC may help to stratify cancer patients, monitor tumor progression as well as response to the therapy.

Recent evidence suggests that angiogenesis is an established hallmark of many solid tumors, while inhibiting proangiogenic factors demonstrates a potential avenue for the treatment [6]. Notably, several studies have proved that angiogenesis results from an imbalance between angiogenic and antiangiogenic factors. Vascular endothelial growth factor (VEGF), as the best known angiogenic factor, promotes proliferation and migration of endothelial cell [7, 8]. In addition, it is also known that angiopoietin-2 (Ang-2), a cytokine upregulated in tumor endothelial cells and some tumor cells including NSCLC, stimulates tumor angiogenesis in collaboration with VEGF and other proangiogenic factors [9].

Angiopoietin-1 (Ang-1) and Ang-2 are ligands for the tyrosine kinase receptor Tie2; they are not only widely expressed on many embryonic tissues, but also expressed on some cancer cells. There is a complicated and homeostatic balance between Ang-1 and Ang-2; some studies suggest that Ang-2, a partial agonist and inhibitor of Ang-1, normally restrains Ang-1-induced activation of the Tie2 receptor [10]. However, other scholars believed that Ang-2 delivers proangiogenic activity through binding the Tie2 receptor with the presence of VEGF [11, 12]. Accumulated evidence shows that the Tie2-signaling pathway is involved in NSCLC and is a potential therapeutic target [13]. For example, AMG 386, neutralizing the interaction between angiopoietins (Ang-1/2) and their Tie2 receptors, inhibits tumor angiogenesis and tumor growth successfully [14, 15].

While the role of Ang-2 in angiogenesis and tumor therapy is well-established, it is also found that Ang-2 expression is associated with prognosis of various tumors, such as chronic lymphocytic leukemia [16], hepatocellular carcinoma [17], colorectal cancer [18], and melanoma [19]. For example, Volkova et al. analyzed Ang-2 of serum samples in colorectal cancer patients (*n* = 344) and confirmed serum Ang-2 as a significant predictor for outcome of colorectal cancer, as well as metastatic CRC treated with bevacizumab-containing therapy [18].

According to available data, previous studies mainly considered that Ang-2 correlated with progression and prognosis of NSCLC significantly. For instance, Coelho et al. [20] detected circulating Ang-2 messenger RNA (mRNA) in NSCLC patients’ blood samples (*n* = 92) and found that a highly circulating Ang-2 mRNA level serving as a significantly unfavorable prognostic factor in NSCLC overall survival is a unique and practical diagnostic tool to determine prognosis in NSCLC. Furthermore, Fawzy et al. [21] suggested that serum angiopoietin-2 is a useful marker for the diagnosis of NSCLC by ELISA technique. Additionally, another study involving 335 unselected stage I-IIIA NSCLC patients described the relationship between the Ang-2 expression and survival; illuminated Ang-4 and Ang-2 were independently associated with survival, and the expression of VEGF was strongly associated with that of Ang-2 [22].

In view of the previous studies, they emphasize that Ang-2 is significantly relevant to the clinical outcome of NSCLC [23, 24]. However, Kabalak et al. [25] used the ELISA method to measure serum VEGF and Ang-2 levels collected from 100 lung cancer patients (87 NSCLC) and then found that VEGF and Ang-2 showed a weak positive correlation; in addition, a higher Ang-2 level in patients may be useful for differential diagnosis, whereas there was no relation between Ang-2 levels and survival days significantly. Reinmuth et al. [26] analyzed tissue samples of 72 patients with primary stages I and II NSCLC and found that neither the expression of Ang-2 nor VEGF was associated with survival.

Although Ang-2 is implicated in prognosis and clinical outcome of NSCLC, other studies did report conflicting results that Ang-2 had no effect on survival. Therefore, it remains unknown whether this discrepancy is caused by limited sample sizes, tumor differentiation, tumor stage, and lymphatic invasion. In this study, a meta-analysis was performed to assess the relationship between Ang-2 expression and prognosis, a clinical outcome of NSCLC.

## Methods

### Search strategy

This meta-analysis was carried out in accordance with the guidelines of the meta-analysis of PRISMA (preferred reporting items for systematic reviews and meta-analyses). In this study, we took a comprehensive search strategy (“angiopoietin-2 OR ANGPT2 protein” and “Carcinoma, Non Small Cell Lung OR Lung Carcinoma, Non-Small-Cell OR Non small Cell Lung Cancer”) through an electronic search on PubMed, Cochrane Library, EMBASE, and CNKI. We also performed a manual search for the articles in the references. Articles, incepted and ended on November 2015, were identified by two investigators (Xuan ZX and Zhang S) independently. Studies included in the meta-analysis had to meet the following criteria: (1) all patients were pathologically diagnosed as NSCLC in clinic; (2) the content of Ang-2 was detected in all patients; and (3) the relationship between the Ang-2 expression and the prognosis of NSCLC patients was investigated. Articles were excluded based on any of the following criteria: (1) duplicated articles or data; (2) no clinical specimens; and (3) abstracts, unpublished studies, reviews.

### Data extraction

Data was extracted independently by two investigators (Xuan ZX and Zhang S), using a standard form. Any discrepancies were solved via discussion, and all the data were subject to consensus. We extracted information including first author’s name, year of publication, country of the study population, case of the study population, type of tumor, stage, analysis method, evaluation method, the follow-up time (months), outcome indexes, and hazard ratio (HR) (95 % CI). Some studies provided HR and *p*; we used the following mathematical formula: b = ln(HR), std = b/inverse_normal_distribution(p/2), 95 % CI = exp(b ± 1.96*std) to calculate the 95 % CI.

### Qualitative assessment

Quality assessment of each available study was performed using the Newcastle–Ottawa Quality Assessment Scale (NOS) for the Cochrane Non-Randomized Studies. Included studies were scored for both bias and NOS independently by two authors (Xuan ZX and Zhang S), and referral to the panel made failing consensus agreement, and a score of 0–9 could be determined to indicate the quality of each study (Table 1). Studies labeled with five or more stars were considered to be of high quality.

**Table 1:** Baseline characteristics of the 20 trials used in the meta-analysis

Author	Year	Country	No. of patients	Stage	Assay	Follow-up (months)	NOS score
Andersen [20]	2011	Norway	335	I–III	IHC	86	8
Coelho [22]	2014	Portugal	92	I–IV	RT-PCR	NA	6
Daly [27]	2014	USA	197	NA	ELISA	60	7
Huang [28]	2012	China	59	I–III	IHC	NA	6
Jiang [29]	2015	China	50	I–III	IHC	NA	5
Li [30]	2015	China	88	I–IV	ELISA	NA	5
Liu [31]	2010	China	50	I–IV	IHC	60	7
Liu [32]	2014	China	40	I–IV	ELISA	30	6
Massabeau [33]	2008	France	78	III	IHC	NA	5
Park [34]	2007	South Korea	110	I–IV	ELISA	22.1	8
Park [35]	2009	South Korea	101	I–II	ELISA	NA	5
Sasaki [36]	2012	Japan	110	I–IV	RT-PCR	60	8
Takanami [37]	2004	Japan	77	I–III	RT-PCR、IHC	60	7
Tanaka [38]	2002	Japan	236	I–III	IHC	60	8
Wan [39]	2008	China	98	I–IV	ELISA	12	5
Wang [40]	2015	China	114	I–IV	IHC	NA	7
Xiao [41]	2010	China	41	I–III	IHC	NA	7
Xing [42]	2003	China	52	I–III	RT-PCR	NA	7
Yuan [43]	2007	China	46	NA	IHC	NA	5
Zhang [44]	2008	China	37	I–III	IHC	NA	7

*NA* not available, *NOS* Newcastle–Ottawa Scale, *RT*-*PCR* real-time polymerase chain reaction, *IHC* immunohistochemistry, *ELISA* enzyme-linked immunosorbent assay

### Statistical analysis

The differences in the studies were shown by the odds ratio (OR) and the corresponding 95 % confidence interval (CI), and no overlap of the 95 % CI with 1 indicated a statistical significance. In order to quantify the aggregation of survival results, HR and their 95 % CI were combined to give the effective value, and HR was calculated from data, which were reported directly or presented in the form of Kaplan–Meier survival curve. In our study, the heterogeneity of included studies was tested by the *I*^2^ statistic based on Cochran’s *Q* test, *I*^2^ < 50 % or *p* > 0.1 was considered to be of low heterogeneity, and the fixed-effect model was used to calculated the pooled OR; otherwise, a random-effect model was used because of the significant heterogeneity. We also evaluated the stability of the results through removing each study at a time. Two-sided *p* < 0.05 was considered statistically significant. All statistical analyses were carried out by STATA 12.0 and Review Manager 5.3.

## Results

### Study characteristics

**Fig. 1:**
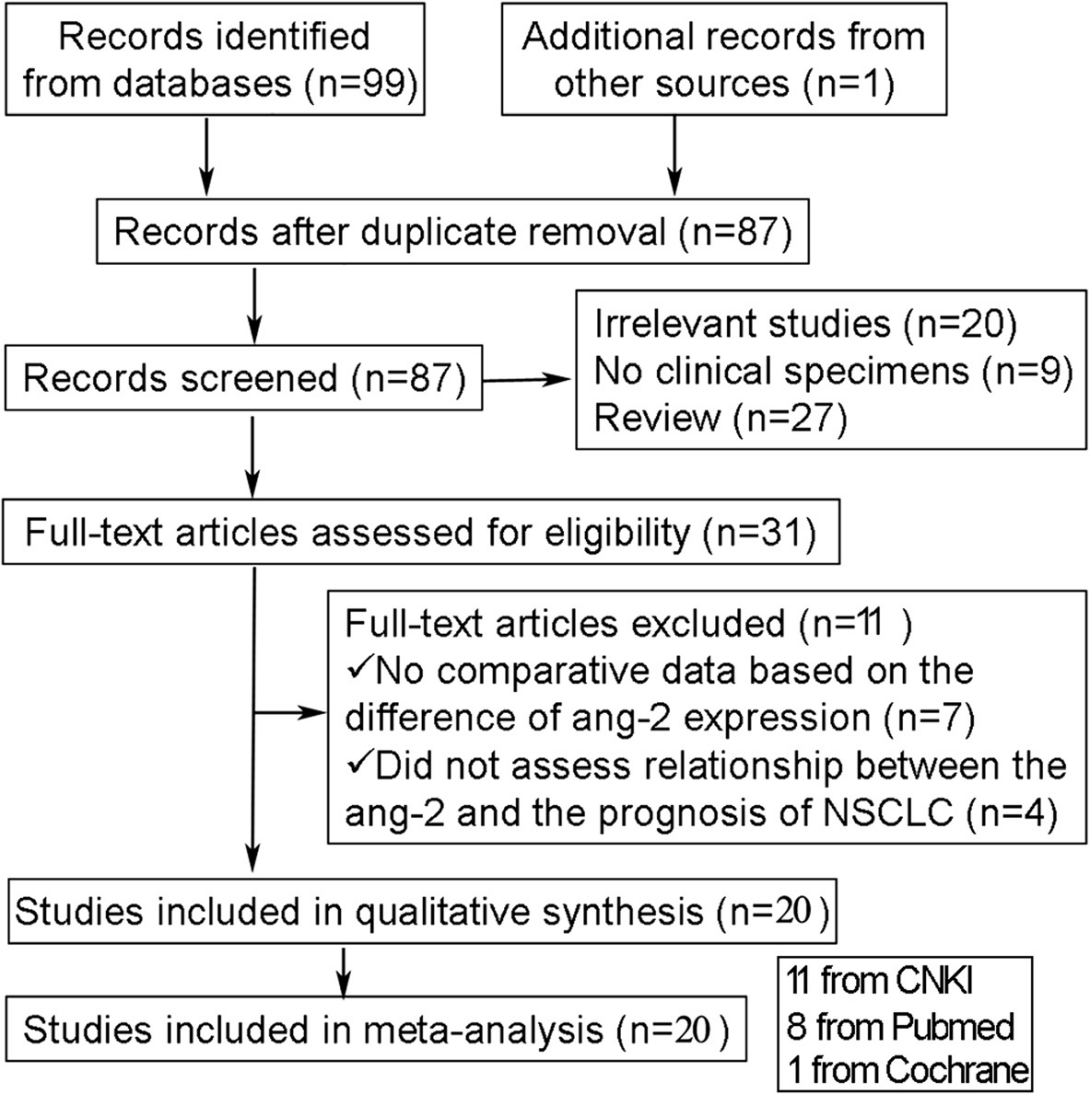
Flowchart showing study selection procedure

The flowchart of the study selection process is presented in Fig. 1. Eleven articles are excluded for no comparative data based on the difference of Ang-2 expression, or no assessment of relationship between the Ang-2 expression and the prognosis of NSCLC. Twenty eligible publications are included in our meta-analysis with 2011 NSCLC patients in total [20, 22, 27–44]. These eligible articles were published from 2002 to 2015. The features of the eligible studies are shown in Table 1. These studies mainly illuminated the correlation between the expression of Ang-2 and NSCLC, based on either prognostic factors or clinicopathological features. One of the 20 studies was from the USA, 11 studies were from China, three studies were from Japan, one study was from France, one study was from Norway, two studies were from South Korea, and one study was from Portugal. Of them, the NOS score is from 5 to 8, so all of the articles were regarded as high quality.

### Different expression of Ang-2 between NSCLC tissues and normal tissues

**Fig. 2:**
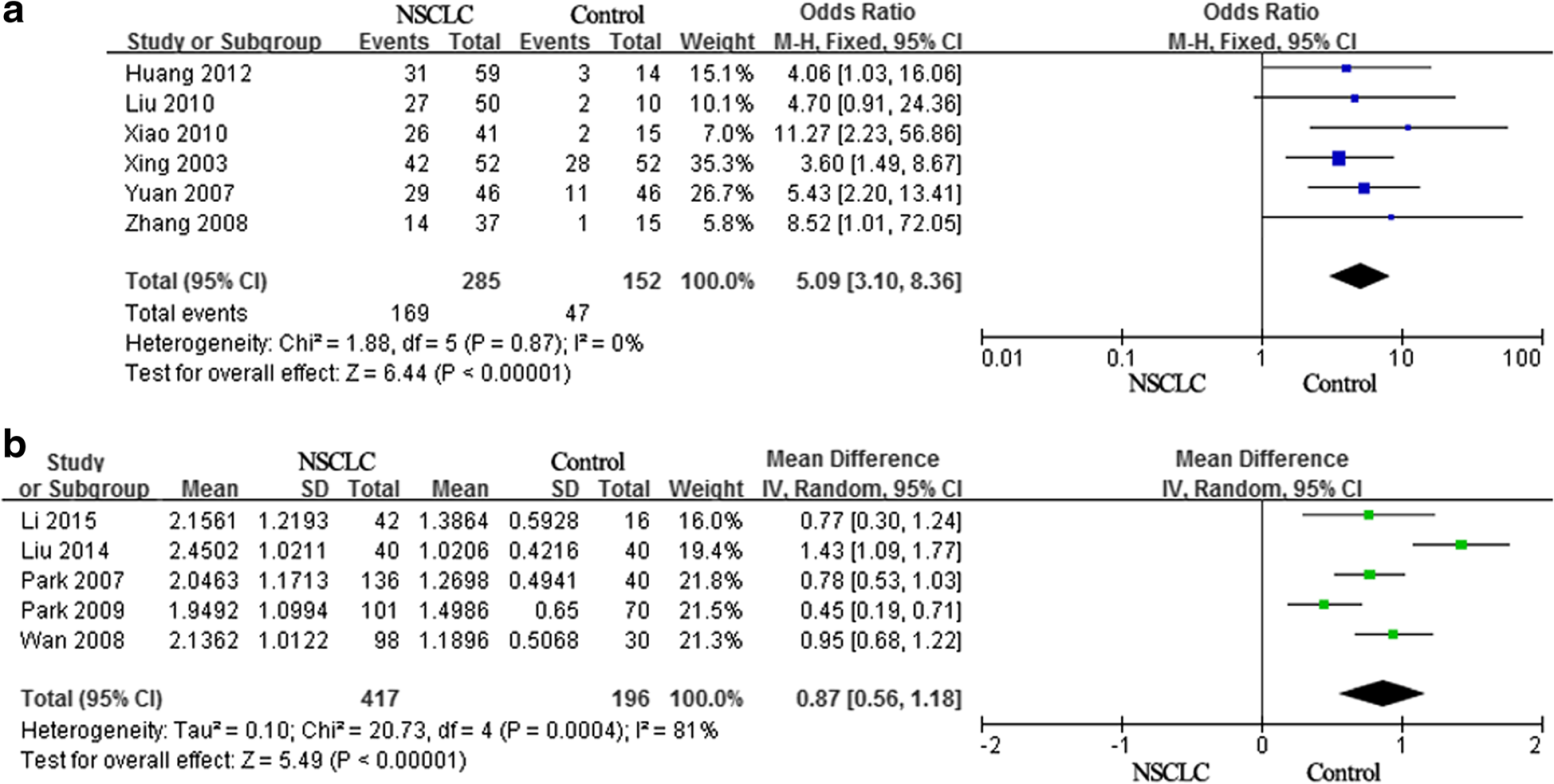
The difference of the Ang-2 expression on NSCLC patients and control. **a** For the meta-analysis of two-category variables, six articles assessed the correlation between Ang-2 expression and NSCLC. **b** Five articles detected Ang-2 content through ELISA in NSCLC serum or normal serum, to analyze the relationship between Ang-2 expression and NSCLC

For the meta-analysis of two-category variables, there were six articles, which assessed the correlation between Ang-2 expression and NSCLC [28, 31, 41–44], our results demonstrated that Ang-2 expressions in NSCLC tissues were significantly higher than normal lung tissues (pooled OR = 5.09, 95 % CI: 3.10–8.36, *p* = 0.000 and *I*^2^ = 0 %; Fig. 2a). In addition, five articles detected Ang-2 through ELISA in NSCLC serum or normal serum, to analyze the relationship between Ang-2 expression and NSCLC [30, 32, 34, 35, 39]. Due to the high heterogeneity among these studies (*P* = 0.000, *I*^2^ = 81 %), we chose the random-effect model. As showed in the Fig. 2b, all the content of Ang-2 in NSCLC tissues was higher than that in normal tissues, and Ang-2 over-expression was associated with NSCLC (control: pooled OR = 0.87, 95 % CI: 0.56-1.18, *P* = 0.000).

### Correlation of Ang-2 expression and clinicopathological features of NSCLC

**Fig. 3:**
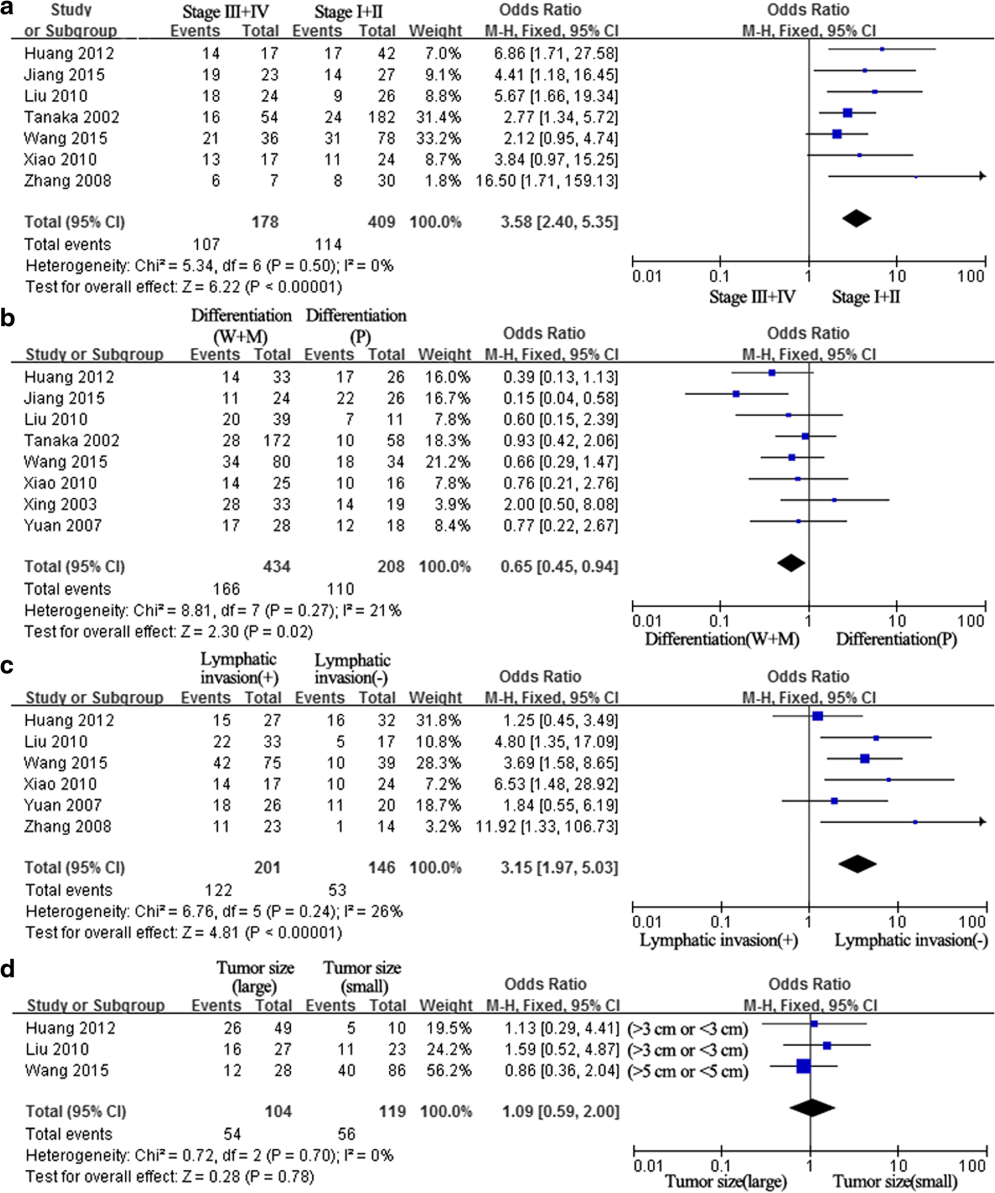
Association of Ang-2 expression with clinicopathological parameters. **a** Seven studies investigated the relationship between Ang-2 expression and tumor stages, and their pooled results revealed that Ang-2 expression significantly associated with tumor stages. **b** Eight studies illuminated the association of Ang-2 expression with tumor differentiation; the combined OR revealed that there was a significant association between Ang-2 expression and tumor differentiation. **c** Ang-2 expression had a significant relationship with lymphatic invasion in NSCLC patients. **d** The combined OR showed that Ang-2 expression was not significantly associated with tumor size

There were seven studies investigating the relationship between Ang-2 expression and tumor stages, TNM stages I and II were considered as low stage and III and IV as high stage [28, 29, 31, 38, 40, 41, 44]. Their pooled analysis revealed that Ang-2 expression significantly associated with tumor stages (pooled OR = 3.58, 95 % CI: 2.40–5.35, *p* = 0.000 and *I*^2^ = 0 %; Fig. 3a). Eight studies illuminated the association of Ang-2 expression with tumor differentiation [28, 29, 31, 38, 40–43]; the combined OR revealed that there was a significant association between Ang-2 expression and tumor differentiation (pooled OR = 0.65, 95 % CI: 0.45–0.94, *P* = 0.02 and *I*^2^ = 21 %; Fig. 3b). The association between Ang-2 expression and lymphatic invasion is illustrated in Fig. 3c. Ang-2 expression had a significant relationship with lymphatic invasion in NSCLC patients [28, 31, 40, 41, 43, 44], with the pooled OR of 3.15 (95 % CI: 1.97–5.03, *p* = 0.000 and *I*^2^ = 26 %). To explore the relevance between Ang-2 expression and tumor size, tumors were classified based on the tumor size (cutoff value was 3 or 5 cm), and three studies were included, and the combined OR showed that Ang-2 expression was not significantly associated with tumor size [28, 31, 40], with a pooled OR estimate of 1.09 (95 % CI: 0.59–2.00, *p* = 0.78 and *I*^2^ = 0 %; Fig. 3d).

### Correlation of Ang-2 expression and survival of NSCLC patients

**Fig. 4:**
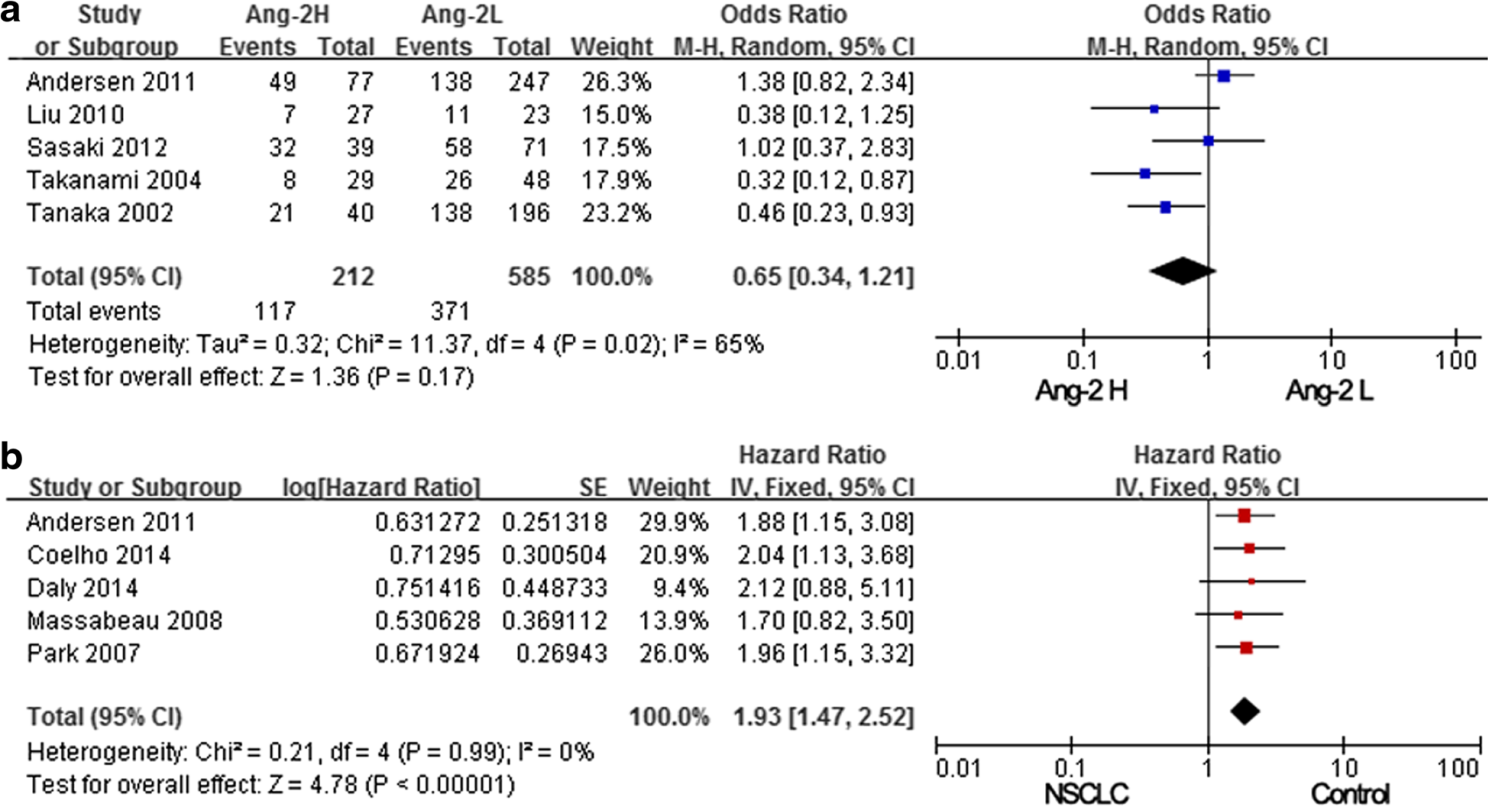
Association of Ang-2 expression with survival. **a** NSCLC patients in five studies were divided into Ang-2 H (high expression) group and Ang-2 L (lower expression) group via the Ang-2 level, and results showed that the overall survival in the Ang-2 L group is tendentiously higher than that of the Ang-2 H group. **b** The pooled data from these five studies suggested that Ang-2 expression was significantly associated with poor survival of NSCLC

The cytoplasmic staining of NSCLC tissues was scored with regard to density as well: 1 = low, 2 = intermediate, and 3 = high, and high expression for Ang-2 was defined as ≥2.5. In our meta-analysis, the NSCLC patients in five studies were divided into the Ang-2 H (high expression) group and the Ang-2 L (lower expression) group via the Ang-2 level, and the overall survival in both groups was calculated [22, 31, 36–38]. The collected data were significantly heterogeneous (*p* = 0.02, *I*^2^ = 65 %). Thus, a random model was used. This meta-analysis showed that the overall survival in Ang-2 L group is tendentiously higher than that of the Ang-2 H group (pooled OR = 0.65, 95 % CI: 0.34–1.21, *p* = 0.17; Fig. 4a).

Five studies provided sufficient information about the correlation of Ang-2 expression and survival of NSCLC patients, especially the hazard ratio (HR) for overall survival (OS) or disease-free survival (DFS) and 95 % confidence interval (CI) [20, 22, 27, 33, 34]. The study of Daly provided HR = 2.12 and *p* = 0.094, we used mathematical formula to calculate the 95 % CI, that was 0.88–5.11. Then, pooled data from these five studies suggested that Ang-2 expression was significantly associated with poor survival of NSCLC (Fig. 4b). There was no significant heterogeneity (*I*^2^ = 0 %, *p* = 0.995), and the pooled HR was 1.93 (95 % CI: 1.47–2.52), indicating that Ang-2 expression predicted worse prognosis in NSCLC.

Sensitivity analysis was carried out to confirm the stability of the result. As shown in Table 2, the pooled HRs ranged from 1.90 (95 % CI: 1.40–2.57, *p* = 0.000) to 1.97 (95 % CI: 1.47–2.63, *p* = 0.000), and relative heterogeneity remained insignificant. Therefore, there was no individual study that could significantly influence the result of HR when any individual study was removed from our meta-analysis.

**Table 2:** Sensitivity analysis for different expressions of Ang-2 between NSCLC tissues and normal tissues

Study omitted	Resulting HR (95 % CI)
None	0.87 (0.56–1.18)
Li 2005	0.89 (0.52–1.25)
Liu 2014	0.73 (0.50–0.96)
Park 2007	0.90 (0.47–1.32)
Park 2009	0.98 (0.69–1.27)
Wan 2008	0.85 (0.44–1.26)

## Discussion

NSCLC is one of common neoplastic diseases, and increasing evidence proves that there is poor prognosis and limited therapeutic options [45]. Therefore, it is urgent to identify reliable biomarkers, which may be helpful in predicting prognosis and guiding surveillance in NSCLC [46]. To our knowledge, tumor angiogenesis, characterized by the formation of new irregular blood vessels derived from preexisting vascular network, plays a central role in NSCLC tumor growth. Recently, the angiopoietins have been shown to be important regulators of neovascularization and endothelial cell survival in malignant tumors [47]. Considered as the major activating ligand to the tyrosine kinase receptor Tie2, Ang-1 usually recruits and sustains peri-endothelial supporting cells to promote endothelial cell survival and vessel stabilization. In general, Ang-2 has been recognized as a naturally occurring antagonist to Ang-1 and prevents Tie2 activation [9, 47]. However, some studies have suggested that Ang-2 can either agonize or antagonize the Tie2 receptor, thereby leading to vascular sprouting or regression [48].

More and more studies indicated that Ang-2 expression is significantly linked with prognosis of NSCLC [22]. Nevertheless, some studies suggested that Ang-2 levels did not correlate with survival days, and the association between Ang-2 and the prognosis of NSCLC remained controversial [25, 26, 49]. Therefore, we conduct this meta-analysis to systematically determine the association of Ang-2 levels with clinicopathological features and prognosis of NSCLC.

Firstly, we assessed the difference of Ang-2 expression between NSCLC tissues and normal tissues. Our result revealed that the expression of Ang-2 in NSCLC tissues was significantly higher than that in normal lung tissues either through two-category variables or continuous variable analysis, and Ang-2 over-expression may be a predictive marker because of the correlation with NSCLC. Then, we also studied the pooled association between Ang-2 expression and clinicopathological features. The pooled data of our results indicated that Ang-2 expression was positively correlated with TNM stages, tumor differentiation, and lymphatic invasion of NSCLC. These results of meta-analysis supported the hypothesis that Ang-2 might contribute to malignant progression of NSCLC, which subsequently leads to a poorer prognosis. Furthermore, the correlation of Ang-2 expression and survival of NSCLC patients was also assessed; the pooled data indicated that Ang-2 expression significantly predicted poor survival (Table 3). Moreover, removing one individual study could not significantly change the linear trend and the stability of the result.

**Table 3:** Sensitivity analysis for correlation of Ang-2 expression and survival of NSCLC patients

Study omitted	Resulting HR (95 % CI)
None	1.93 (1.47–2.52)
Andersen 2011	1.95 (1.41–2.69)
Park 2007	1.92 (1.40–2.62)
Massabeau 2008	1.97 (1.47–2.63)
Coelho 2014	1.90 (1.40–2.57)
Daly 2014	1.91 (1.44–2.53)

## Conclusions

In conclusion, we conducted a systematical and comprehensive meta-analysis to assess the relationship between Ang-2 expression and prognosis, a clinical outcome of NSCLC, and the prognostic significance of Ang-2 expression for NSCLC was identified by comparing the depth of tumor stages, tumor differentiation, lymphatic invasion, and other clinicopathological features. Eventually, our data showed that Ang-2 expression is significantly linked with poor prognosis for patients with NSCLC.

However, it was shown that Ang-2 expression did not significantly link with tumor size, but there were only three studies included, and tumors divided into two portions based on tumor size were 3 or 5 cm, so the research about relationship between tumor size and Ang-2 expression should be studied in the future. Additionally, current data about Ang-2 expression and NSCLC prognosis is limited, and some studies containing negative conclusions were not published. Only large studies illuminating the prognostic value of Ang-2 in NSCLC substantiated our conclusions; Ang-2 expression may act as a potential predictor for prognosis of patients with NSCLC. Ang-2 will provide useful information for a therapeutic target to treat NSCLC at the moment.

## Abbreviations

NSCLC: Non-small cell lung cancer
Ang-2: Angiopoietin-2
Ang-1: Angiopoietin-1
EGFRs: Epidermal growth factor receptors
ALK: Anaplastic lymphoma kinase
NOS: Newcastle–Ottawa Quality Assessment Scale
OR: Odds ratio
95 % CI: Corresponding 95 % confidence interval
DFS: Disease-free survival
HR: Hazard ratio
